# Deciding on Optical Illusions: Reduced Alpha Power in Body Dysmorphic Disorder

**DOI:** 10.3390/brainsci12020293

**Published:** 2022-02-21

**Authors:** Anastasios E. Giannopoulos, Ioanna Zioga, Konstantinos Kontoangelos, Panos Papageorgiou, Fotini Kapsali, Christos N. Capsalis, Charalabos Papageorgiou

**Affiliations:** 1 School of Electrical & Computer Engineering, National Technical University of Athens, 15780 Athens, Greece; ccaps@central.ntua.gr; 2 Donders Institute for Brain, Cognition and Behaviour, Radboud University Nijmegen, 6525 EN Nijmegen, The Netherlands; joannazioga@gmail.com; 3 First Department of Psychiatry, National and Kapodistrian University of Athens Medical School, Eginition Hospital, 11528 Athens, Greece; kontoangel@med.uoa.gr; 4 Department of Electrical and Computer Engineering, University of Patras, 26334 Patras, Greece; papageorgiou@ece.upatras.gr; 5 Psychiatric Hospital of Attica, 16121 Athens, Greece; fotini_kapsali@hotmail.com; 6 Neurosciences and Precision Medicine Research Institute “Costas Stefanis”, University Mental Health, 11527 Athens, Greece; chpapag@med.uoa.gr

**Keywords:** body dysmorphic disorder, EEG, optical illusions, alpha oscillations

## Abstract

Background: Body dysmorphic disorder (BDD) is a psychiatric disorder characterized by excessive preoccupation with imagined defects in appearance. Optical illusions induce illusory effects that distort the presented stimulus, thus leading to ambiguous percepts. Using electroencephalography (EEG), we investigated whether BDD is related to differentiated perception during illusory percepts. Methods: A total of 18 BDD patients and 18 controls were presented with 39 optical illusions together with a statement testing whether or not they perceived the illusion. After a delay period, they were prompted to answer whether the statement was right/wrong and their degree of confidence in their answer. We investigated differences of BDD patients on task performance and self-reported confidence and analyzed the brain oscillations during decision-making using nonparametric cluster statistics. Results: Behaviorally, the BDD group exhibited reduced confidence when responding incorrectly, potentially attributed to higher levels of doubt. Electrophysiologically, the BDD group showed significantly reduced alpha power at the fronto-central and parietal scalp areas, suggesting impaired allocation of attention. Interestingly, the lower the alpha power of the identified cluster, the higher the BDD severity, as assessed by BDD psychometrics. Conclusions: Results evidenced that alpha power during illusory processing might serve as a quantitative EEG biomarker of BDD, potentially associated with reduced inhibition of task-irrelevant areas.

## 1. Introduction

Body dysmorphic disorder (BDD) is an often-severe psychiatric disorder, recently classified within the Obsessive-Compulsive and Related Disorders (OCD) [1]. BDD is characterized by excessive preoccupation with imagined defects in appearance that are not at all or slightly observable to others (DSM-5) [2]. Constant preoccupation is associated with many time-consuming rituals, such as mirror gazing or constant checking [1]. Optical illusions are context-induced subjective distortions of visual features, such as the length, size, shape, or direction of elements within a visual context [3,4,5]. Notably, it is widely suggested that optical illusions induce illusory effects that distort the presented stimulus thus leading to ambiguous percepts [5,6]. An individual might perceptually experience an illusion despite them being aware of the illusory feature, suggesting a dissociation between perceptual and conceptual knowledge [7]. Nevertheless, perception of illusions constitutes a crucial feature of our visual system, for example, by perceiving the world as stable even when we are moving [8]. Therefore, visual illusions might prove valuable for studying not only vision processing, but also higher-order cognitive processes [8,9].

In many cases, BDD patients show differentiated visual processing [10]. For instance, BDD patients exhibited diminished inversion effect during face processing [11], as well as increased perception of changes in facial features [12] compared to controls, suggesting greater detailed/local than global processing. However, besides enhanced processing of details, BDD patients perceived non-existing distortions when looking at images of their own faces [13]. Furthermore, BDD patients considered faces of others as being angry when they were not, suggesting an increased difficulty in interpreting facial expressions [14]. Considering the visual particularities of BDD patients in their own face processing as well as the subjective nature of visual illusory perception, here we examined the susceptibility of BDD patients to visual illusions (i.e., illusory percepts that are not related to the self) and the respective brain activation during decision-making on the illusions.

The overarching question of our study is whether BDD is related to differentiated perception of optical illusions and brain activation while making judgements on the illusions. Do BDD patients succumb to illusory effects more than healthy people do? Are BDD patients less confident when making judgements on optical illusions? What are the neural correlates of the decision-making process, and, finally, are those related to BDD severity? We investigated these questions by presenting BDD patients and healthy controls with optical illusions and asking them to make subjective judgements, while the electroencephalogram (EEG) was recorded.

A promising way to investigate the neurophysiological signatures of BDD patients would be to consider their observed cognitive deficits. Previous studies have shown that BDD patients exhibit executive dysfunction, as evidenced from impairments in memory and attention in the digit span, story memory recall, and Stroop interference tasks [15]. BDD and OCD patients show significantly poorer memory and attention compared to healthy controls, as assessed by the Repeatable Battery for the Assessment of Neuropsychological Status (RBANS) [10]. During processing of inverted faces on a screen, BDD patients show a reduced inversion effect compared to healthy controls, attributed to greater focus on detail (over-attention) and reduced holistic processing [11]. Furthermore, OCD is characterized by high levels of doubt, uncertainty, and repetitiveness [16,17]. For instance, in checking situations, OCD sufferers are doubtful of whether or not they performed the ritual and need to repeat it over and over again [18].

Previous research investigating the neural signatures of BDD in various tasks has interpreted the results mainly on the basis of the attentional impairments that this patient group exhibits [19,20,21,22,23]. It is known that OCD patients, including BDD, are constantly preoccupied with a certain action/feature and are not able to suppress their obsessive thoughts [1]. Furthermore, constant preoccupation is linked to time-consuming rituals, such as skin picking, mirror gazing, grooming, and checking [1]. Constant checking of the perceived flaw in mirrors in BDD has been associated with cognitive dysfunction of the inhibition of unwanted impulses, as in OCD [24]. In this context, alpha oscillations have been thought to fine-tune sensory processing by actively inhibiting task-irrelevant networks [25,26]. In other words, alpha oscillations facilitate information processing by the mechanisms of so called “gated inhibition”, during which brain networks that are irrelevant for given task purposes are actively suppressed by functional inhibition associated with increased alpha power [25]. Alpha power increase has been suggested to reflect active processing of task-relevant stimuli simultaneously with suppression of regions not required for the task [26,27]. In line with this framework, decreased alpha power during cognitive tasks in BDD and OCD patients has been associated with their inability of cognitive inhibition [21]. More specifically, previous studies reported reduced task-related alpha power in OCD patients compared to healthy controls [20,22]. Min and colleagues (2011) [20] used a color and shape discrimination task, while Simpson and colleagues (2000) [22] exposed subjects to OCD symptom-provoking stimuli. Furthermore, in two recent studies, our group found reduced alpha power at the left temporo-parietal areas [19] in response to prepulse inhibition in BDD patients compared to healthy controls, attributed to impaired resource allocation.

Following up from the above, the present study aims to investigate the neural correlates of BDD by analyzing brain oscillations during decision-making on optical illusion judgements. We examined this by presenting BDD patients and healthy controls with optical illusions, while recording their EEG. After a delay period has passed, they were prompted to make a judgement on the illusion, showing whether they had succumbed or not to the illusory effect. They were also asked to report the degree of confidence for their answer. Participants completed questionnaires assessing BDD symptomatology. On the behavioral level, we investigated whether BDD influenced performance on the task and self-reported confidence, whereas, from the electrophysiological perspective, we analyzed the brain oscillatory activity during the decision-making period. Finally, we examined the relationship between BDD severity and the identified neural signatures.

Based on the aforementioned studies on cognitive deficits in BDD [11], we expect that BDD patients will not show impaired performance in identifying the illusory effects, as the current evidence suggests over-attention and non-holistic processing in BDD patients only for their own face processing. It is also expected that BDD patients will report higher uncertainty about their answers, in line with enhanced feelings of doubt that characterize this disorder [28]. We further hypothesize that BDD patients will show reduced alpha oscillatory activity during decision-making, potentially attributed to impaired inhibition of task-irrelevant thoughts. Finally, we will explore what brain signatures are related to BDD degree of symptomatology.

## 2. Materials and Methods

### 2.1. Participants

Thirty-six adult volunteers took part in this study. Eighteen patients comprised the body dysmorphic (BDD) group (9 females, mean ± SD age of 36.7 ± 8.3 years; 9 males, mean ± SD age of 27.1 ± 6.0 years). Eighteen healthy controls (CTL) were matched for age and sex (10 females, mean ± SD age of 28.8 ± 4.9 years; 8 males, mean ± SD age of 30.0 ± 5.7 years). The absence of significant group differences in age was confirmed by an independent-samples *t* test (t(34)=−1.081,p=0.287). Participants underwent clinical interviews by two psychiatrists. BDD was diagnosed according to the DSM-5 criteria. The YBOCS-BDD Questionnaire and the Dysmorphic Concern Questionnaire confirmed the diagnosis. All participants had no history of neurological or psychiatric disorders, and normal or corrected vision.

The study was conducted in the psychophysiology laboratory of the University Mental Health, Neurosciences and Precision Medicine Research Institute “Costas Stefanis” (U.M.H.R.I.), in collaboration with the First Department of Psychiatry, Medical School, Eginition Hospital, National and Kapodistrian University of Athens. All participants were informed about the experimental procedure and provided written consent prior to their participation. The study was approved by the local ethics committee of the First Department of Psychiatry, Medical School, Eginition Hospital, National and Kapodistrian University of Athens.

### 2.2. Experimental Design and Procedure

**Figure 1:**
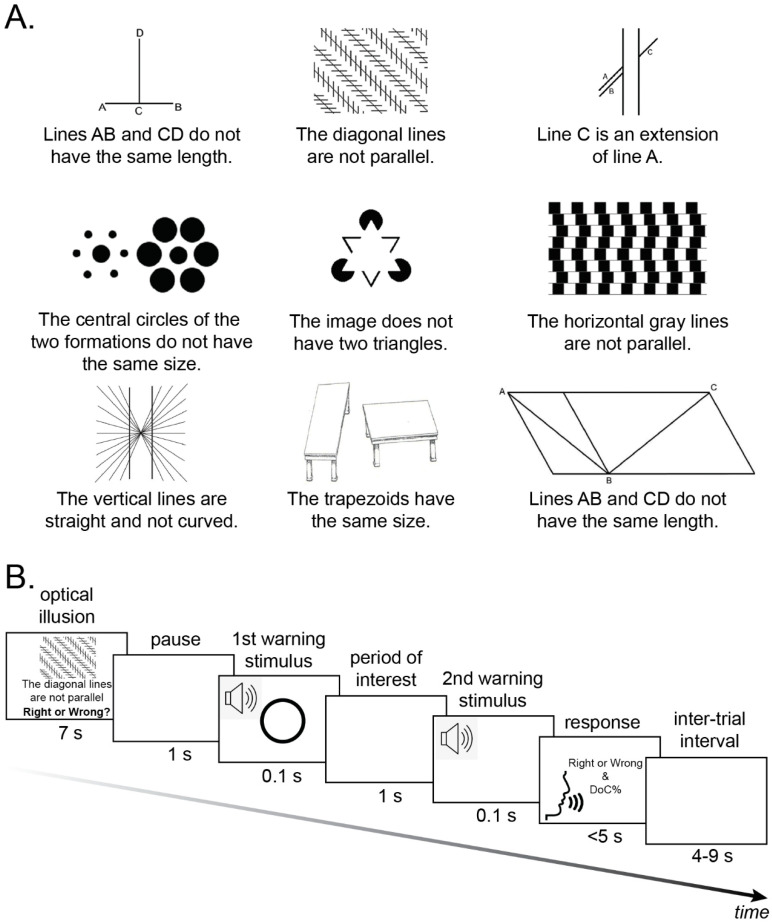
(**A**) Examples of the experimental stimuli composed by Papageorgiou and colleagues (2020) [6]. The illusions depicted are the following: vertical-horizontal, Zöllner, Poggendorff, Ebbinghaus, Kanizsa’s triangle, café wall, Hering, Shepard tables, and Sander. The images have been obtained by the authors from Wikipedia website where they were made available under a CC BY-SA 3.0 license; (**B**) Illustration of the trial structure. First, an optical illusion is presented on screen for 7 s, together with a statement and the question Right or Wrong? below the stimulus. A blank screen is then presented for 1 s, followed by a 0.1 s warning stimulus tone. Participants are given 1 s to think of their response. A second warning stimulus tone is then presented for 0.1 s. Afterwards, participants verbalize two responses: 1. Whether the statement is right or wrong, and 2. What is their degree of confidence (DoC) for their answer. There is an inter-trial interval jittered from 4 to 9 s.

We used a set of 39 optical illusions composed by Papageorgiou and colleagues (2020) [6] (see Figure 1A for examples). Those comprised two-dimensional images (28 black and white, 11 colored) of 23 well-known optical illusions plus their variations. The length of the images ranged from 8 to 33 cm, while the height of the images ranged from 5.5 to 14.5 cm. The optical illusions were accompanied with written statements referring to a feature of the respective image. More specifically, there were 19 right (e.g., illusion 3 in Figure 1A: Line C is an extension of line A) and 20 wrong statements (e.g., illusion 2 in Figure 1A: The diagonal lines are not parallel).

Participants were seated in a Faraday cage to minimize interference caused by external electromagnetic fields during the EEG recording. They were asked to look straight and keep their eyes open throughout the session. Participants were instructed that they would be presented with 39 optical illusions together with a written statement, and would be prompted to answer two questions: 1. Whether the statement is right or wrong, and 2. What is their degree of confidence for their answer on a scale from 0 (not at all certain) to 100 (absolutely certain). In each trial, the optical illusion was presented on screen for 7 s (see Figure 1B for the trial structure). A statement referring to the illusion was presented below the stimulus, as well as the question Right or Wrong? A blank screen was then presented for 1 s, followed by a 0.1s warning stimulus tone (500 Hz, 65 dB). Then, participants were given 1 s to think of their response. A second warning stimulus tone was then presented for 0.1 s prompting participants to respond. Participants’ verbal responses were recorded by an experimenter seated outside the testing room. There was an inter-trial interval jittered from 4 to 9 s.

### 2.3. EEG Recording and Preprocessing

The EEG signals were recorded from 30 Ag/AgCl electrodes mounted on an elastic cap according to the International 10–20 System: Fp1, F3, P3, O1, F7, T3, T5, AFz, Fz, FCz, CP3, FC3, TP7, Fpz, FT7, Oz, FT8, Fp2, F4, C4, P4, O2, F8, T4, T6, Cz, Pz, CPz, CP4, FC4. The sampling frequency was 1 kHz. Electrode impedance was kept constantly below 5 kΩ. EEG activity was referenced online to the average of the left and right ear lobes, while the ground electrode was placed on the left mastoid.

The continuous EEG data of each subject were high-pass filtered at 1 Hz to remove DC offsets and baseline drifts. The data were then low-pass filtered at 45 Hz. Using the “clean_rawdata” function, an EEGLAB [29] plug-in for bad channel detection, along with visual inspection of the detected bad channels, electrodes showing abnormal time-course were excluded and interpolated. EEG signals were then re-referenced to the whole-scalp common average. Subsequently, an Independent Component Analysis (ICA) of the continuous and event-marked EEG data was performed to correct eye-blinks and saccades. To semi-automatize the process of hand-labeling artifactual components, rejection was performed by visual inspection along with simultaneous consideration of the SASICA tool [30]. In controversial cases, we consulted the MARA tool suggestions [31]. The SASICA guidelines were parameterized as: “Autocorrelation” (Threshold r = auto; Lag = 20 ms), “Focal components” (Threshold z = auto), “Correlation with EOG” (enabled for VEOG and HEOG with threshold r = 0.2), “ADJUST” [32] and “FASTER” [33] methods (enabled for blink channels). Both software tools automatically labeled artifactual components, which were suggested for rejection candidates. The final rejection decision was jointly evaluated based on the (i) MARA/SASICA-driven suggestions and visual inspection of the (ii) components’ topography (blink artifacts showed frontal distribution; saccade artifacts exhibited frontal and anti-hemispherical distribution) and (iii) spectra (spectrum with local skewness, outliers and/or steep curves were rated as artifacts). The average number of rejected components per group was 3.4 ± 1.1 and 3.2 ± 1.2 for CTL and BDD, respectively. Finally, continuous data were epoched from −0.5 to 1.0 s around the first warning stimulus tone.

### 2.4. Psychometric Ratings

The Y-BOCS and the DCQ questionnaires were used in order to investigate potential correlations between BDD symptomatology and EEG measures.

- *Yale-Brown Obsessive-Compulsive Scale (Y-BOCS) for BDD*: This psychometric questionnaire evaluates the severity of BDD symptoms [34]. We used a 12-item version translated, adapted and validated in Greek [35]. Items 1–5 assess obsessional preoccupation with the perceived defect in appearance, while items 6–10 assess compulsive behaviors. Item 11 measures the degree of insight, and item 12 avoidance. It is rated on a 0 (not at all) to 4 (every day) Likert scale. Scores for all items are summed up to create the total score.
- *Dysmorphic Concern Questionnaire (DCQ)*: This questionnaire is a 7-item self-report measure that assesses cognitive and behavioral symptoms of physical overconcern without seeking to establish a “diagnosis” of BDD [36]. Respondents rate their concern on their physical appearance on a 4-point scale, ranging from 0 (not at all) to 3 (much more than most people).

Independent samples *t* tests were used to confirm the differences in psychometrics between control vs. BDD (DCQ: t(34)=−10.9,p<0.001; Y-BOCS: t(34)=−13.8,p<0.001). The descriptive statistics for control vs. BDD psychometrics, respectively, are 5.9 ± 0.8 vs. 18.9 ± 0.8 (in DCQ) and 3.7 ± 0.6 vs. 29.8 ± 1.7 (in Y-BOCS).

### 2.5. Data Analysis

Non-parametric statistics were only used to compare the spatiotemporal (channel- and time-specific) EEG responses, since the need to correct for multiple comparisons was pronounced. On the contrary, behavioral data comparisons between the two groups were performed via parametric statistics.

#### 2.5.1. Behavioral Analysis

***Correctness*:** First, we assessed participants’ ability to escape from the illusory effect of the images by evaluating their responses in the first question (*Right or Wrong?*). Specifically, a response was considered correct if the subject did not succumb to the illusory effect, while it was considered incorrect if the subject succumbed to the illusion. For each participant, correctness was calculated as the number of their correct responses divided by the total number of trials (39). To investigate whether BDD influenced the perception of illusions, we performed an independent samples *t* test between percentage correct of the CTL vs. the BDD group.

***Degree of Confidence (DoC)*:** To examine whether correctness influenced the level of confidence in the two groups, a 2 (*correctness*: *correct* vs. *incorrect*) × 2 (*group*: *CTL* vs. *BDD*) mixed ANOVA was performed on their DoC, averaged over the respective trials.

#### 2.5.2. EEG Analysis

***Time-frequency representation (TFR)*:** To analyze oscillatory brain activity during the period of interest (decision-making), we conducted a time-frequency analysis from −0.3 to 0.8 s time-locked to the onset of the first warning tone. Specifically, the preprocessed continuous time-series of each channel (concatenated across trials) was convolved with complex Morlet wavelets using 50 linearly separated frequencies (from 1 to 40 Hz) and a variable number of wavelet cycles (from 3 to 12). Convolution was performed via multiplication between the spectra of kernel and EEG trials. The final time-frequency power spectra of each channel were calculated as the average time-frequency power spectra across trials to obtain a final time-frequency representation (TFR) with increased signal-to-noise ratio. Single-subject TFRs (power values, P) were dB-normalized ($P_{dB}$) based on the pre-stimulus period from −0.3 to −0.1 s by applying the following formula (for each time-frequency point (t,f)):

(1) $$P_{dB}\left(t,f\right)=10\cdot \log_{10}\frac{P\left(t,f\right)}{\frac{1}{200}\sum_{i\in \left[-0.3,-0.1\right]}P\left(i,f\right)}$$

The power of each frequency band was then calculated by averaging the power values within delta (1–4 Hz), theta (4–8 Hz), alpha (8–12.5 Hz), beta (12.5–30 Hz), and gamma (30–40 Hz) bands. Electrode- and band-specific time-courses (power values relative to pre-stimulus baseline) were calculated for each participant.

***Nonparametric cluster permutation test*:** We used a nonparametric cluster permutation procedure to compare the alpha oscillatory power during the period of interest in CTL vs. BDD groups. All time points were considered from 0 to 0.8 s around the period of interest. First, all the possible uncorrected *t* values (resulting from independent samples *t* tests) were computed for each electrode and time-point, and absolute *t* values smaller than 2 were discarded. Then, clusters of spatiotemporally neighboring *t* scores were formed. The clustering process for any pair of the remaining *t* scores was based on three criteria, according to whether same-sign *t* values are neighbored in space and time. Specifically, any pair (i,j) of *t* values belongs to the same cluster if and only if:

(1) *i* and *j* belongs to neighboring electrodes
(2) *i* and *j* belongs to successive time-points
(3) *i* and *j* have the same sign.

Subsequently, empirical distribution curves of the group differences were estimated using 5000 random permutations by shuffling the subject labels (CTL vs. BDD). In each randomly permuted instance, we calculated the sum of *t* values within each cluster. Then, the maximum (absolute value) cluster score was considered as the cluster *t*-statistic. All randomizations were conducted for a rejection of the null hypothesis and a control of false alarm rate at p=0.05 (two-tailed). Clusters formed by the actual labels with a *t* value exceeding the *t* critical values acquired from the permutation analysis were finally identified. As a control analysis, the same statistical procedure was conducted also for the other bands (delta, theta, beta, and gamma).

#### 2.5.3. Relationship between EEG Measures and BDD Severity

We examined potential relationships between the EEG clusters identified from the nonparametric cluster permutation procedure and BDD severity. For this purpose, we computed the Pearson’s product-moment correlation coefficients between the identified cluster and the psychometric indices of BDD severity, as assessed by the DCQ and Y-BOCS ratings, separately.

## 3. Results

### 3.1. Behavioral Results

**Figure 2:**
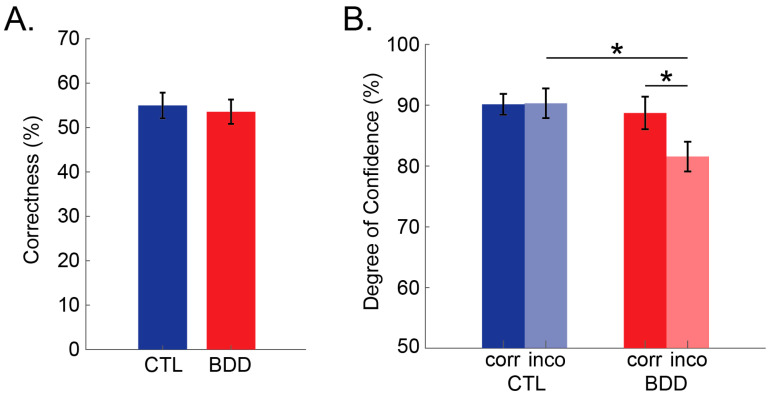
Behavioral performance on the optical illusion task. (**A**) Percentage of correct responses (i.e., identifying the illusory effect) separately for the control (CTL; blue) and the body dysmorphic group (BDD; red); (**B**) Degree of confidence separately for CTL and BDD when responding correctly (opaque) and when responding incorrectly (transparent). Error bars represent ±1 SEM. * *p* < 0.050.

***Correctness*:** Independent samples *t* tests showed no significant differences in correctness between the two groups (p=0.722). Specifically, the average percentage of correct responses for the CTL group was 55.0 ± 12.2%, whereas for the BDD group correctness was at 53.6 ± 11.6% (Figure 2A).

***Degree of Confidence (DoC)*:** A 2 *(correctness*: *correct* vs. *incorrect)* × 2 *(group*: *CTL* vs. *BDD)* mixed ANOVA revealed that participants were more confident of their answers when they responded correctly (M=89.437, SE=1.587) compared to when they responded incorrectly (M=85.934, SE=1.726) (main effect of *correctness* ($F\left(1,34\right)=5.501,\;p=0.025,\;\eta_{p}^{2}=0.139$). Results also showed a significant *correctness* × *group* interaction ($F\left(1,34\right)=6.026,\;p=0.019,\;\eta_{p}^{2}=0.151$). To investigate the interaction further, planned contrasts were conducted. Interestingly, the BDD group exhibited significantly lower DoC when responding incorrectly compared to when responding correctly (t(17)=2.727, p=0.014), whereas the CTL group showed no significant difference in DoC between conditions (p=0.910). Finally, the BDD group showed significantly lower DoC than the CTL group for incorrect answers (t(34)=2.537, p=0.016); however, that was not the case for correct answers (p=0.657) (Figure 2B).

### 3.2. EEG Results

**Figure 3:**
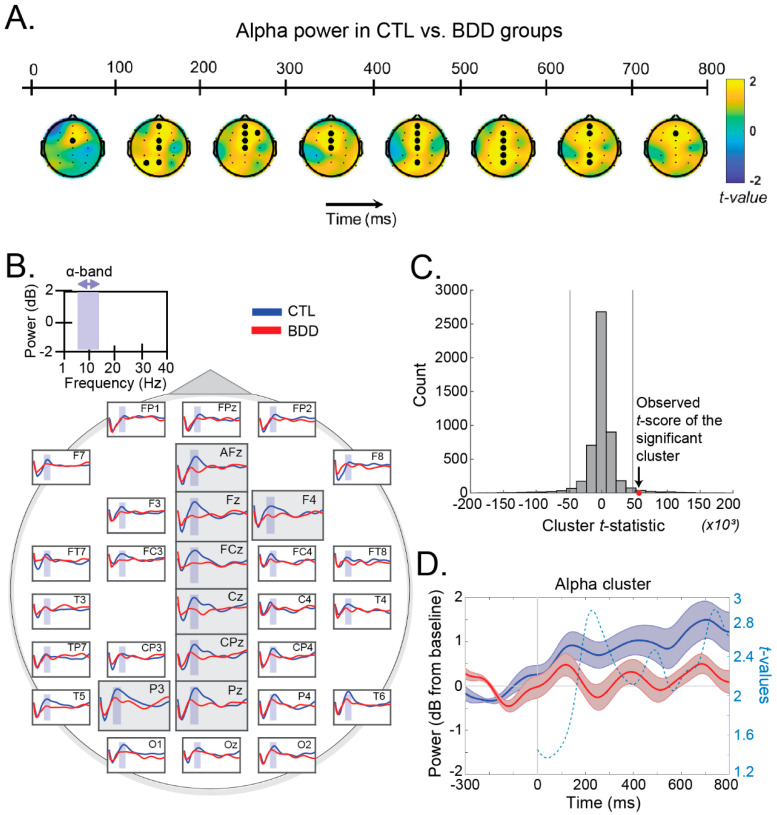
(**A**) Topographical maps of the *t*-values between the control (CTL) vs. the body dysmorphic patients (BDD) group in the alpha frequency band from 1–800 ms (in steps of 100 ms). The significant cluster consists of electrodes AFz, FCz, F3, Fz, FCz, Cz, CPz, Pz and P3. In each time window, electrodes that are significant for at least 70% of the time are highlighted in bold; (**B**) Scalp array of the group-specific (blue: CTL; red: BDD) grand-average spectrum. Purple-shaded range corresponds to the alpha frequency band (8–12.5 Hz); (**C**) Difference distribution (CTL vs. BDD) for the cluster *t*_max-statistic in the alpha band. The red marker corresponds to the *t*-statistic of the significant spatiotemporal cluster; (**D**) Grand-average waveforms of the alpha activity for CTL (blue) and BDD (red) groups over the significant cluster (averaged across the electrodes of the cluster). Shaded areas correspond to ±1 SEM. The dashed line indicates the time-course of the *t*-values (right *y*-axis).

The cluster permutation procedure revealed a significant spatiotemporal cluster in the alpha band, showing greater power in the CTL than the BDD group ($t=56.8\times 10^{3}$) (Figure 3C). Specifically, electrodes AFz, Fz, F4, FCz, Cz, CPz, Pz, and P3 exhibited higher alpha power in CTL compared to BDD subjects (Figure 3B). The topographical maps of the *t* values in the alpha band in successive 100 min time windows are presented in Figure 3A. Control analysis revealed no significant clusters in the delta, theta, beta, or gamma bands.

No significant clusters were identified in the rest of the bands, namely delta (1–4 Hz), theta (4–8 Hz), beta (13–30 Hz), and gamma (30–45 Hz).

### 3.3. Relationship between EEG Measures and BDD Severity

**Figure 4:**
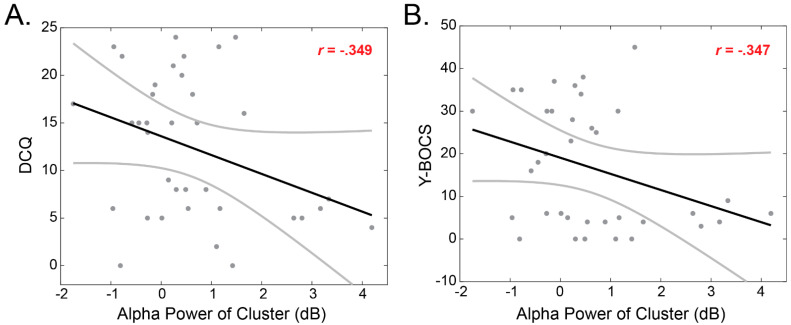
Relationship (Pearson’s coefficients) between the alpha power of the fronto-central/parietal cluster of electrodes and BDD questionnaires. (**A**) Correlation between alpha power and the DCQ questionnaire; (**B**) Correlation between alpha power and the Y-BOCS questionnaire. Gray lines illustrate 95% confidence interval.

Significant outcomes resulting from the prior behavioral and electrophysiological analyses were further assessed for potential correlational relationships. Firstly, the alpha power of the significant cluster (averaged across the electrodes) was calculated for each participant and for possible correlation with psychometric ratings. Correlation was assessed via Pearson’s product-moment coefficients. Results showed that the alpha cluster was positively correlated both with the Y-BOCS (*r* = −0.347, *p* = 0.038) and the DCQ scores (*r* = −0.349, *p* = 0.037) (Figure 4).

No significant linear relationships were identified between the alpha power and the behavioral responses. Specifically, the alpha power averaged across the electrodes identified in Section 3.2 was not correlated with Correctness (*r* = −0.6, *p* = 0.34), Confidence in correct answers (*r* = 0.08, *p* = −0.62), or Confidence in incorrect answers (*r* = 0.24, *p* = 0.15).

## 4. Discussion

In this study, we investigated the neural correlates of BDD patients relative to healthy controls during decision-making on judgements of optical illusions. In brief, results showed no differences in performance accuracy between BDD and CTL groups, i.e., groups were equally correct in identifying illusions. Interestingly, the BDD group exhibited lower confidence than the CTL group when responding incorrectly. Time-frequency analysis showed that BDD patients exhibit reduced alpha power, primarily at the fronto-central and parietal areas. Finally, the lower the alpha power of the identified cluster, the higher the BDD severity.

The first hypothesis, that the BDD group would not show poorer performance in identifying illusory features than the CTL group, was supported. There were no significant differences between BDD and CTL groups in the percentage of correct responses. There could be a few explanations for this finding. First, it is possible that BDD patients show impaired performance when making judgements on their own appearance, while their performance is unaffected when judging external, non-threatening stimuli. For instance, during the processing of inverted faces on a screen, BDD patients show reduced inversion effect compared to healthy controls, attributed to a greater focus on detail (over-attention) and reduced holistic processing [11]. Second, it could be that the task was very easy and might not have provided sufficient challenge to distinguish performance between groups. However, the behavioral results do not suggest a ceiling effect, which speaks against this possibility. Previous research on visual perception in BDD shows contradictory findings. In particular, BDD patients show better processing of facial features and details, suggesting increased local compared to global processing [12]. However, this has been disputed in studies showing no differences between BDD patients and controls in the Navon task and other face processing tasks [37]. Considering that faces are processed differently than other objects (Bate et al., 2019), BDD might be associated with differences in face processing rather than visual processing in general. In support of this, a meta-analysis demonstrated differences in higher-order processing in BDD patients rather than effects on local visual processing [38], and specifically increased selective attention towards threats as well as memory abnormalities.

Our second hypothesis with regards to the BDD group exhibiting lower confidence was supported. Specifically, the BDD group was less confident of their responses than the CTL group, but only when they were incorrect. This is in line with the observation that OCD is characterized by high levels of doubt and uncertainty [28,39], potentially reflecting an inability to remember or monitor previous actions [40]. Tolin and colleagues (2001) [18] measured memory accuracy and confidence in OCD in a memory recall task of objects. Interestingly, OCD patients reported lower confidence in their memories when repeatedly exposed to threat-related objects [18]. However, there was no difference in memory accuracy between OCD patients and controls. Results suggested that lower confidence in OCD is not due to memory deficits. Rather, the authors proposed that it might reflect the increased doubt observed in OCD patients when the same ritual is performed over and over again [18].

On the neural level, BDD patients showed reduced alpha power at the fronto-central and parietal scalp areas during decision-making compared to healthy controls. Alpha oscillations are principally associated with the regulation of attentional processes [41]. In particular, alpha oscillations have been shown to facilitate resource allocation to task-relevant brain areas, by inhibiting task-irrelevant areas [25]. This is achieved in a top-down manner by guiding attention, suppressing distracting input, and facilitating the processing of task-relevant stimuli [25]. Aligned with this, reduced alpha band activity has been related to difficulties in inhibiting task-irrelevant distractors [42]. For instance, Haegens and colleagues (2011) [43] found that lateralization of alpha power positively influenced the subjects’ behavioral performance on a spatial discrimination task, i.e., both accuracy and reaction times improved with the degree of alpha lateralization. Further, Pogarell and colleagues (2006) [44] found reduced alpha power during the wakeful-resting condition in OCD patients compared to healthy controls. In the context of illusory perception, alpha oscillations have been proved to play a critical role, mainly reflecting the degree of excitability [45]. Specifically, increased excitability is associated with low alpha power. BDD patients have impaired attentional processes, potentially associated with a hyperactivity of the functional circuits involved in the selective attention [46,47]. Therefore, our findings may indicate an over-attention of BDD patients during illusory perception.

Interestingly, we found that alpha power was negatively correlated with BDD severity. Previous neurophysiological research has identified neural indices of perceptual distortions in BDD [48,49]. For example, Scholz and colleagues (2017) [50] found reduced N170 amplitude in BDD patients during visual processing of faces and houses. Frontostriatal hyperactivity has been mainly associated with obsessive thoughts and compulsive behaviors, as evidenced in an fMRI study [49]. Furthermore, the brain anatomical characteristics of BDD patients revealed correlations between BDD symptom severity and volumes of the left inferior frontal gyrus and right amygdala, potentially contributing to the involvement of these regions in pathological face processing [51]. Our study might add to the previous findings by proposing alpha oscillations as a novel biomarker of illness phenotype to be used in clinical practice.

This research is not without limitations. First, it might be that different neural processes take place when participants succumb to the illusory effect vs. when they do not succumb to the effect. However, the low number of trials did not allow comparisons for brain activity for correct vs. incorrect responses. Second, due to the low signal-to-noise ratio of the EEG data, it was not possible to further investigate the neural signatures of confidence in the single-trial level. It is noteworthy that this study focused only on task-related alterations between the BDD and control groups. Based on evidence indicating resting-state differences in OCD [44], additional research is also needed to shed light on the resting-state potentials of BDD and clarify whether the latter show distinct patterns beyond task-related paradigms. It should be also noted that the absence of OCD traits recording in the study population defines a limitation in interpreting the increased uncertainty of BDD as directly equivalent with the doubtfulness of OCD patients. Although BDD and OCD show similarities in compulsive behaviors, their symptomatology and pathophysiology signature are non-identical. To that end, additional research is needed to investigate whether the existing knowledge of OCD can be generalized in BDD and vice versa. Furthermore, we acknowledge that the exact time point of decision-making cannot be estimated with high accuracy in decision-making tasks with a fixed response time such as ours. However, considering that participants were clearly instructed on the time window of decision-making before the initiation of the experiment and that it would require more cognitive effort to maintain a decision in working memory if that was taken earlier, we can be confident that decision-making took place as instructed. Non-invasive brain stimulation experiments might also be useful to provide causal evidence for the role of alpha oscillations during optical illusion processing, by directly stimulating participants at that frequency band. Finally, the absence of reaction times recording sets another limitation in this study, since an additional index of the required reaction time for decision-making in BDD would allow for joint evaluation of both the uncertainty level and the response delay.

## 5. Conclusions

In the present study, we investigated the electrophysiological correlates of BDD patients relative to healthy controls during decision-making on judgements of optical illusions. The obtained results showed no differences in performance accuracy between BDD and CTL groups, i.e., groups were equally correct in identifying illusions. However, the BDD group demonstrated inferior confidence than the CTL group when responding incorrectly. Time-frequency analysis revealed that BDD patients exhibit reduced power in alpha brain oscillations, primarily at the fronto-central and parietal areas. Notably, the lower the alpha power of the identified cluster, the higher the BDD severity. In conclusion, we provide evidence that the alpha oscillatory power during illusory processing might serve as a quantitative EEG index for BDD, potentially associated with reduced inhibition of task-irrelevant areas.

## Data Availability

Data supporting the findings of this study are available from the corresponding author, upon reasonable request.
